# Molecular Free Electron Vortices in Photoionization by Polarization-Tailored Ultrashort Laser Pulses

**DOI:** 10.3389/fchem.2022.899461

**Published:** 2022-06-02

**Authors:** Tim Bayer, Matthias Wollenhaupt

**Affiliations:** Ultrafast Dynamics Group, Institut für Physik, Carl von Ossietzky Universität Oldenburg, Oldenburg, Germany

**Keywords:** free electron vortices, polarization-tailored femtosecond laser pulses, diatomic molecules, multiphoton ionization, coupled electron-nuclear dynamics, polarization-shaped bichromatic fields, photoelectron momentum distribution

## Abstract

Atomic and molecular free electron vortices (FEVs), characterized by their spiral-shaped momentum distribution, have recently attracted a great deal of attention due to their varied shapes and their unusual topological properties. Shortly after their theoretical prediction by the single-photon ionization (SPI) of He atoms using pairs of counterrotating circularly polarized attosecond pulses, FEVs have been demonstrated experimentally by the multiphoton ionization (MPI) of alkali atoms using single-color and bichromatic circularly polarized femtosecond pulse sequences. Recently, we reported on the analysis of the experimental results employing a numerical model based on the *ab initio* solution of the time-dependent Schrödinger equation (TDSE) for a two-dimensional (2D) atom interacting with a polarization-shaped ultrashort laser field. Here, we apply the 2D TDSE model to study molecular FEVs created by SPI and MPI of a diatomic molecule using polarization-tailored single-color and bichromatic femtosecond pulse sequences. We investigate the influence of the coupled electron-nuclear dynamics on the vortex formation dynamics and discuss the effect of CEP- and rotational averaging on the photoelectron momentum distribution. By analyzing how the molecular structure and dynamics is imprinted in the photoelectron spirals, we explore the potential of molecular FEVs for ultrafast spectroscopy.

## 1 Introduction

Vortex beams, such as optical vortices (Babiker et al., 2002; Shen et al., 2019; Eickhoff et al. 2020b) and electron vortex beams (Verbeeck et al., 2010; Lloyd et al., 2017), which are characterized by their helical wave fronts, are currently attracting much attention both theoretically and experimentally. Recently, the analogies between free electron vortices (FEVs) in transmission electron microscopy and multiphoton ionization (MPI) have been pointed out (Eickhoff et al., 2020c). In photoionization, FEVs are created by ionizing a quantum system with tailored ultrashort circularly polarized laser pulse sequences. Introducing a time-delay *τ* between two counterrotating circularly polarized subpulses results in an “unusual kind of Ramsey interference” Ngoko Djiokap et al. (2015), which gives rise to an azimuthal interference pattern in the photoelectron momentum distribution (PMD) forming a multi-armed Archimedean spiral. Atomic FEVs have been studied theoretically employing different numerical methods, including the *ab initio* solution of the time-dependent Schrödinger equation (TDSE) (Ngoko Djiokap et al. 2015; Ngoko Djiokap et al., 2016; Djiokap and Starace., 2017; Ngoko Djiokap et al., 2017; Ivanov et al., 2017; Jia et al., 2019; Xiao et al., 2019; Bayer et al., 2020; Qin et al., 2020; Wang et al., 2020; Zhen et al., 2020; Geng et al., 2020; Maxwell et al., 2020; Djiokap et al., 2021; Geng et al., 2021), calculations based on the strong-field approximation (Hasović et al., 2016; Ivanov et al., 2017; Li M. et al., 2017; Li et al., 2018a; Li et al., 2018b; Gazibegović-Busuladžić et al., 2018; Kong et al., 2018; Li et al., 2019b; Xiao et al., 2019; Geng et al., 2020; Maxwell et al. 2020; Qin et al., 2020; Geng et al., 2021; Becker and Milošević, 2022), perturbative analytical approaches (Ngoko Djiokap et al., 2015; Ngoko Djiokap et al., 2016; Djiokap and Starace, 2017; Ngoko Djiokap et al., 2017; Djiokap et al., 2021), emerging techniques such as the R -matrix with time-dependence theory (Clarke et al., 2018; Armstrong et al., 2019; Maxwell et al., 2020) and semi-classical Monte-Carlo methods (Ben et al., 2020). Current research topics comprise the creation of FEVs with odd rotational symmetry using bichromatic pulse sequences (Ngoko Djiokap et al., 2016), the study of electron-electron correlations in atomic double-ionization (Djiokap and Starace, 2017; Ngoko Djiokap et al, 2017), the influence of the AC-Stark effect on FEVs from strong-field ionization (Kong et al., 2018; Li et al., 2019a), the application of FEVs to probe electron displacement in strong-field ionization (Xiao et al., 2019), the detection of ring currents (Wang et al., 2020), the interferometric use of FEVs for quantum phase retrieval Qin et al. (2020) and unusual subjects such as multiphoton pair-production in single-color (Li Z. L. et al., 2017) and bichromatic (Li Z. L. et al., 2018) fields. A comparison between FEVs characterized by their spiral-shaped PMD and free electron vortex states (Bliokh et al., 2017) [also termed twisted electrons (Maxwell et al., 2020)] derived from the fluid-dynamical formulation of quantum mechanics (Madelung, 1927) is given in (Geng et al., 2020; Geng et al. 2021; Kerbstadt et al., 2020).

The first experimental demonstration of atomic FEVs was reported in (Pengel et al., 2017b). By MPI of potassium atoms with *single-color* time-delayed counterrotating circularly polarized pulse sequences, we observed FEVs with four-, six and eight-fold rotational symmetry (Pengel et al., 2017a; Pengel et al., 2017b). Subsequently, we demonstrated the creation of seven-fold rotationally symmetric FEVs by MPI of sodium atoms (Kerbstadt et al., 2019b) using time-delayed *bichromatic* sequences. In the same work, FEVs with odd rotational symmetry have been created using bichromatic fields in the limit *τ* → 0. In addition, we reported on crescent-shaped FEVs (Kerbstadt et al., 2019b; Eickhoff et al., 2020a; Eickhoff et al., 2021c) and a five-fold symmetric FEV (Eickhoff et al., 2021c) created by bichromatic MPI. In the strong-field ionization regime, Mancuso *et al.* observed crescent-shaped and three-fold symmetric FEVs using co- and counterrotating bichromatic fields, respectively (Mancuso et al., 2015, Mancuso et al., 2016). Similarly, Eckart *et al.* created three-fold symmetric FEVs (Eckart et al., 2016; Eckart et al., 2018) and measured the PMD using cold target recoil-ion momentum spectroscopy (Dörner et al., 2000; Ullrich et al., 2003). In our experiments, we applied photoelectron tomography (Wollenhaupt et al., 2009b; Wollenhaupt et al., 2013) to reconstruct the 3D PMD of the atomic FEVs (Pengel et al., 2017a; Pengel et al., 2017b; Kerbstadt et al., 2019b; Eickhoff et al., 2021c). Recently, this method has been developed further into a holographic scheme to reconstruct the photoelectron wave function by measuring interferograms in the PMD (Eickhoff et al., 2020a; Eickhoff et al., 2021b; Eickhoff et al., 2021c).

Meanwhile, the theoretical research focus has evolved from atomic FEVs towards the investigation of FEVs created on molecular systems. Bandrauk and coworkers were the first to recognize the potential of molecular FEVs as spectroscopic tools sensitive to the molecular geometry and ultrafast electron dynamics by investigating the MPI of $H_{2}^{+}$ (Yuan et al., 2016) and $H_{3}^{2+}$ (Yuan et al., 2017) in co- and counterrotating circularly polarized bichromatic fields. Subsequently, Ngoko-Djiokap *et al.* reported on molecular FEVs in the double-ionization of H_2_ created by single-photon ionization (SPI) (Djiokap et al., 2018) and resonance-mediated two-photon ionization (Djiokap and Starace, 2021). In an initial study of molecular FEVs in the tunneling regime, created by counterrotating circularly polarized bichromatic fields, Ke *et al.* reported on highly structured photoelectron holograms—commonly recorded using linearly polarized pulses (Huismans et al., 2011; Bian and Bandrauk, 2012)—which yielded rich information about the molecular structure and laser-driven electron dynamics (Ke et al., 2019). Spiral-shaped *nuclear* momentum distribution analogous to those of FEVs have been obtained for the fragments of the $H_{2}^{+}$ molecular ion after dissociation along different interfering pathways induced by a single circularly polarized laser pulse (Chen et al,. 2020; Chen and He, 2020). Using co- and counterrotating circularly polarized pulses sequences, Shu *et al.* recently exerted control on the interference of degenerate Zeeman states in the $H_{2}^{+}$ molecular ion, mapped into the PMD by an ionizing third pulse (Shu et al., 2020). Very recently, Guo *et al.* presented a numerical study on N_2_ based on the *ab initio* solution of the two-dimensional (2D) TDSE, in which they studied the sensitivity of molecular FEVs to the laser parameters (Guo et al., 2021).

**FIGURE 1:**
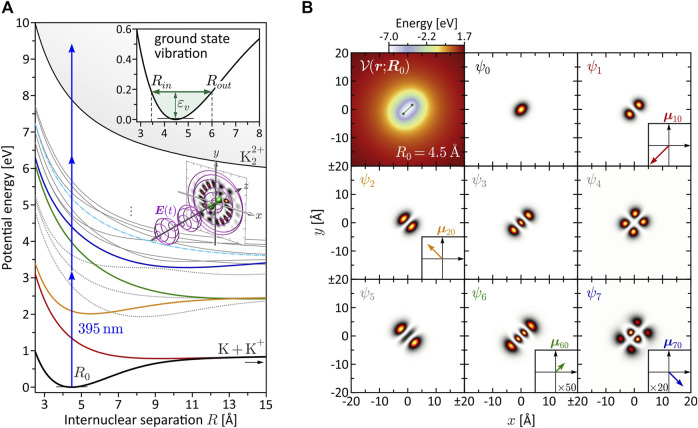
(Color online.) Characterization of the bound molecular system. **(A)** BO potential energy curves *V*_*n*_(*R*). The equilibrium distance in the electronic ground state is found at *R*_0_ = 4.48 Å. Among the eight lowest states, only *V*_1_(*R*) (red), *V*_2_(*R*) (yellow), *V*_6_(*R*) (green) and *V*_7_(*R*) (blue) are dipole-coupled to the ground state. These states are relevant for the investigated MPI schemes. Especially state *V*_6_(*R*) plays a prominent role in the dynamic three-photon ionization scenario (see Section 3.2.2), because it becomes near-resonant with a pulse of central wavelength *λ*_0_ = 395 nm at the outer turning point *R*_*out*_ = 6.0 Å of the vibration (see inset). **(B)** Molecular potential V(r;R) and first eight electronic eigenfunctions *ψ*_*n*_ (***r***; ***R***) for *R* = *R*_0_. The eigenfunctions are used to determine the transition dipole moments ***μ***_*n*0_, coupling the states *ψ*_*n*_ (*n* ≥ 1) to the ground state *ψ*_0_, and to calculate the bound state population dynamics in the MPI scenarios.

Building on our recent numerical study on atomic FEVs (Bayer et al., 2020), here we apply our 2D TDSE model to numerically investigate molecular FEVs created by photoionization of a diatomic molecular ion with polarization-tailored laser pulses. Using polarization-shaped pulses, at least 2D numerical simulations are required to describe the generally asymmetric polarization profiles. In many cases, the 2D approach is sufficient to capture the essential features of the PMD. Compared to a full 3D calculation, 2D simulations benefit from a greatly reduced computational cost. Recently, we employed the 2D TDSE model to accurately reproduce atomic FEVs created experimentally by MPI of alkali atoms (Bayer et al., 2020) to validate our numerical approach. Considering a molecule aligned in the laser polarization plane, the 2D model is expected to yield similarly realistic results for molecular FEVs. The central inset to Figure 1A illustrates the 2D laser-molecule interaction by showing a counterrotating circularly polarized pulse sequence impinging on a diatomic molecule aligned in the laser polarization-plane along with the created 2D PMD. Compared to a full 3D calculation, the polar information of the PMD is lost. However, in the case of FEVs, this information is generally less relevant, since the characteristic spiral pattern is an azimuthal feature of the PMD which is fully contained in a central section parallel to the polarization plane. We note that, although the description in 2D “flatland” is considered an approximation of real space, 2D approaches are relevant for confined condensed matter systems such as surfaces, interfaces or monolayers of e.g., graphene (Novoselov et al., 2012) or transition-metal dichalcogenides (Li et al., 2022). In this contribution, we focus on three aspects of molecular FEVs which are relevant for the design of future experiments and provide insights into the rich variety of physical mechanisms behind the generation of molecular FEVs by polarization-shaped pulses. First, we compare the PMD from different ionization regimes, i.e., SPI vs. MPI, at a range of fixed internuclear separations. Besides the influence of the increased angular momentum transfer in MPI, we investigate to which extend intermediate resonances are involved in the formation of the FEVs. Second, we identify the fingerprints of the coupled electron-nuclear dynamics in the interference structures of the FEVs. To this end, we compare the PMDs from photoionization of a rigid and a vibrating molecule and study the interaction between nuclear motion and electronic ionization dynamics. Specifically, we discuss the role of transient electronic resonances arising during the vibration for FEVs created by MPI and examine signatures of non-adiabatic bound state dynamics in the PMD. Third, we investigate molecular FEVs in the molecular frame vs. laboratory frame by considering molecular rotation and optical phase averaging. We analyze the sensitivity of the PMD to the experimental parameters and define optimal conditions to observe the molecular FEVs in the experiment.

The paper is organized as follows. After the introduction of the numerical model and the characterization of the molecular system in Section 2, we start in Section 3.1 by investigating a rigid molecule interacting with laser pulses of different polarization in the SPI and MPI regime. In Section 3.2, we consider a vibrating molecule and focus on the creation of molecular FEVs *via* SPI and MPI in the presence of coupled electron-nuclear dynamics. Section 3.3 addresses different experimental averaging mechanisms which we discuss on a molecular FEV from bichromatic two- vs. three-photon ionization. In Section 4, we conclude the paper and give a brief outlook.

## 2 Physical System

In this section, we provide the theoretical background to describe the interaction of a diatomic molecule with a polarization-shaped laser field in two dimensions, and present the numerical methods used to solve the 2D TDSE. The 2D TDSE model is based on established numerical techniques (Tannor, 2007; Bauer, 2017) and has been described in more detail in Bayer et al. (2020) for the generation of atomic FEVs. Here we focus on the extension of the model from an atomic to a molecular system. In particular, we describe our semi-classical approach to incorporate the vibrational nuclear dynamics into the 2D TDSE model, in order to investigate the interplay between the nuclear motion and the electronic excitation, i.e., the coupled electron-nuclear dynamics.

### 2.1 Theoretical Description

We consider the interaction of a diatomic molecular ion with a polarization-shaped ultrashort laser pulse propagating in the *z*-direction. The two atoms are arranged in the *x*-*y*-plane which coincides with the laser polarization plane. Their nuclei are separated by ***R*** = *R* ***e***_*R*_, with ***e***_*R*_ being a unit vector parallel to the internuclear axis. Throughout this paper, we consider a homonuclear molecule. The *x*-*y*-coordinate frame is chosen such that $e_{R}=\left(e_{x}+e_{y}\right)/\sqrt{2}$ is aligned along the positive diagonal and the nuclei are situated at ± ***R***/2. Furthermore, we assume a single active valence electron. The molecular system is described by the screened Coulomb potential introduced by Sprik and Klein (1988), Shin and Metiu (1995), Shin and Metiu (1996), Erdmann et al. (2004).

$$V\left(r;R\right)=-\frac{e^{2}}{4\pi \varepsilon_{0}}\left[\frac{z\;erf\left(|r+R/2|/a\right)}{\left|r+R/2\right|}+\frac{z\;erf\left(|r-R/2|/a\right)}{\left|r-R/2\right|}-\frac{z^{2}}{R}\right],$$ (1)

with ***r*** = (*x*, *y*) and erf denoting the error function. Motivated by our recent experiments on potassium atoms (Pengel et al., 2017b) and dimers (Bayer et al., 2013), we choose an effective nuclear charge of *z* = 0.9085 and a softcore parameter of *a* = 2.3065 Å (Eickhoff et al., 2021c) to mimic the singly charged potassium dimer $K_{2}^{+}$ for the study of molecular FEVs. Figure 1A shows the first 15 Born-Oppenheimer (BO) potential energy curves *V*_*n*_(*R*) obtained by solving the time-independent Schrödinger equation

$$\left[-\frac{\hbar^{2}}{2m_{e}}\Delta +V\left(r;R\right)\right]\psi_{n}\left(r;R\right)=V_{n}\left(R\right)\;\psi_{n}\left(r;R\right),$$ (2)

with *m*_*e*_ being the mass of the electron, for different values of *R*. The higher-lying Rydberg states are mainly characterized by Coulomb repulsion between the two nuclei and converge to the repulsive double-ionization potential *V*_*i*_(*R*) (thin black line). The BO potentials aid us in the design of specific excitation scenarios and, in particular, serve to identify intermediate resonances encountered along the MPI pathways. The color coding among the lowest eight states *ψ*_*n*_ indicates their dipole-coupling to the electronic ground state *ψ*_0_. States plotted as gray dotted lines are not coupled to the ground state. The transition dipole moments ***μ***_*n*0_ = −*e*⟨*ψ*_*n*_| ***r*** |*ψ*_0_⟩, with the electron charge −*e*, are determined using the electronic eigenfunctions *ψ*_*n*_ (***r***; ***R***). The eigenfunctions *ψ*_*n*_ (***r***; ***R***_0_) at the equilibrium distance *R*_0_ = 4.48 Å are shown in Figure 1B, along with the molecular potential $V\left(r;R_{0}\right)$ . The insets depict the non-vanishing transition dipole moments; their *R*-dependence is shown in Figure 6C and addressed in Section 3.2.1. In addition to the determination of dipole-couplings, the eigenfunctions are used to calculate the bound state population dynamics *p*_*n*_(*t*) = |⟨*ψ*_*n*_|*ψ*(*t*)⟩|^2^ (see Figure 4) in order to unravel the ionization dynamics in the various MPI scenarios.

The temporal laser electric field is described in the spherical basis (Wollenhaupt et al., 2009a; Bayer et al., 2019; Kerbstadt et al., 2019b). We consider a bichromatic double pulse sequence of the general form (Eickhoff et al., 2021a; Eickhoff et al., 2021c).

$$E^{+}\left(t\right)=E_{1}\;g\left(t-\tau_{1}\right)\;e^{i\left(\omega_{1}t+\varphi_{1}+\varphi_{ce}\right)}\;e_{q_{1}}+E_{2}\;g\left(t-\tau_{2}\right)\;e^{i\left(\omega_{2}t+\varphi_{2}+\varphi_{ce}\right)}\;e_{q_{2}}.$$ (3)

Each subpulse *n* = 1, 2 is characterized by an individual peak amplitude $E_{n}$ , time-delay *τ*_*n*_, central frequency *ω*_*n*_, relative phase *φ*_*n*_ and polarization state $e_{q_{n}}$ (*q*_*n*_ = ±1). The latter can either be left-handed circularly polarized (LCP), described by $e_{1}=-\left(e_{x}+ie_{y}\right)/\sqrt{2}$ , or right-handed circularly polarized (RCP), described by $e_{-1}=-\left(e_{x}-ie_{y}\right)/\sqrt{2}$ . Common to both subpulses is the carrier-envelope phase (CEP) *φ*_*ce*_ and the Gaussian-shaped envelope *g*(*t*) with unit amplitude and a duration of Δ*t* (full width at half maximum of the intensity). In the single-color schemes discussed in Sections 3.1, 3.2, we use *ω*_0_ ≔ *ω*_1_ = *ω*_2_ as central frequency and *λ*_0_ = 2*πc*/*ω*_0_ as the corresponding central wavelength. The real-valued field is given by $E\left(t\right)=R\left[E^{+}\left(t\right)\right]$ .

The laser-molecule interaction is described in the dipole approximation. Initially, we consider a rigid molecule with fixed internuclear separation ***R***. As a consequence, the nuclear kinetic energy vanishes. The corresponding TDSE for the wave function *ψ*(***r***; ***R***, *t*) of the valence electron in the length gauge reads

$$i\hbar \frac{\partial }{\partial t}\psi \left(r;R,t\right)=\left[-\frac{\hbar^{2}}{2m_{e}}\Delta +V\left(r;R\right)+e\;r\cdot E\left(t\right)\right]\psi \left(r;R,t\right).$$ (4)

The TDSE is solved numerically using established methods described in Section 2.2. The *ab initio* TDSE calculation inherently includes all intermediate states, which are relevant for the MPI schemes discussed in Sections 3.2.2, 3.3. Therefore, no further approximations concerning the electronic structure of the molecule are required, as confirmed in Bayer et al. (2020). In our model, the electron dynamics is treated quantum mechanically, whereas the slower dynamics of the nuclei is treated classically, which is an established strategy to reduce the dimensionality of the calculation (Carrasco et al., 2022). Specifically, we model the vibrational nuclear dynamics by inserting a time-dependent function *R* = *R*(*t*) into the electronic potential V[r;R(t)] , which periodically modulates the potential along the internuclear axis. The function *R*(*t*) is obtained by solving the Newtonian equation of motion $\mu \ddot{R}=-dV_{0}/dR$ for the ground state BO potential *V*_0_(*R*). The reduced mass *μ* of the nuclear system serves us as a parameter to design specific vibration scenarios for our studies of coupled electron-nuclear dynamics in Section 3.2. In particular, *μ* is adapted such that the vibrational period *T*_*v*_ is compatible with the employed laser pulses, i.e., in the order of several times the pulse duration Δ*t*. In the experiment, the duration and timing of the pulses need to be adapted to the molecular dynamics (Bayer et al. 2013). The vibrational energy is set to *ɛ*_*v*_ = 180 meV. As depicted in the inset to Figure 1A, this vibrational excitation corresponds to a mildly anharmonic nuclear oscillation between *R*_*in*_ = 3.45 Å (inner turning point) and *R*_*out*_ = 6.0 Å (outer turning point). The choice of the vibrational energy is a trade-off between the amplitude and the period of the oscillation. The vibration amplitude should be sufficiently large to energetically disentangle the photoelectron signals generated at the inner and outer turning point (cf. Section 3.2.1). This condition sets a lower limit on *ɛ*_*v*_. On the other hand, the vibration period should not be too large, otherwise the radial interference pattern of the FEVs created by a sequence of two time-delayed laser pulses locked to different turning points (cf. Section 3.2.2) becomes too dense to be resolved energetically. This condition sets an upper limit on *ɛ*_*v*_. The chosen value of *ɛ*_*v*_ = 180 meV was found to be suitable for all vibration scenarios discussed in Sections 3.2, 3.3.

### 2.2 Numerical Methods

The TDSE in Eq. 4 is solved numerically on a discrete 2D spatial grid. The wave function of the active electron is propagated in time according to

$$\psi \left(r;R,t+\delta t\right)=e^{-i\frac{\delta t}{\hbar }H\left(r;R,t\right)}\psi \left(r;R,t\right),$$ (5)

where H(r;R,t) is the Hamiltonian, written in square brackets on the right-hand side of Eq. 4. The propagator is calculated using a Fourier-based split-operator technique (Feit et al., 1982; Bandrauk and Shen, 1993; Rice and Zhao, 2000; Wollenhaupt et al., 2005; Tannor, 2007; Bauer, 2017; Grossmann, 2018). The time propagation is performed in two consecutive stages. The first stage, starting at the initial time *t*_*i*_ < 0 and ranging up to *T* = −*t*_*i*_, is the interaction with the laser pulse centered around *t* = 0. In this stage, the photoelectron wave packets are created. The second stage ranging up to the final time *t*_*f*_ is the field-free propagation of the wave packets under the influence of the long-ranged Coulomb-type molecular potential. Throughout both stages, we use the same temporal step size of *δt* = 10 as. The wave function is propagated on a square spatial grid with boundaries (*x*_max_, *y*_max_) = −(*x*_min_, *y*_min_) = (500, 500) Å. The spatial resolution is chosen to be *δx* = *δy* = 1 Å. The molecule is initiated in the electronic ground state *ψ*(***r***; ***R***, *t*_*i*_) = *ψ*_0_ (***r***; ***R***). Starting from the solution of the time-independent Schrödinger equation Eq. 2, obtained on a smaller spatial grid using the Fourier grid Hamiltonian method (Marston and Balint-Kurti, 1989), the ground state wave function is refined by imaginary-time propagation (Tal-Ezer and Kosloff, 1986). In order to minimize unphysical reflections of the wave function at the spatial boundaries, we use absorbing boundary conditions (Kosloff and Kosloff, 1986; Santra, 2006) implemented by adding an artificial spherically symmetric imaginary potential $U\left(r\right)\propto -i\;r^{16}$ to the molecular potential V(r;R) in Eq. 1. After the interaction with the laser pulse, the wave function is propagated until the free photoelectron wave packets have detached from the bound part, which remains localized at the two nuclei, but not yet reached the absorbing boundaries. Then the free part of the electron wave function is separated from the bound part by application of a spherically symmetric splitting filter of the form $f\left(r\right)=1-e^{-\ln\left(2\right){\left(\frac{r}{\Delta r}\right)}^{4}}$ , similar to Heather and Metiu (1987). The PMD P(k) is proportional to the modulus square of the momentum space wave function ${\tilde{\psi }}_{f}\left(k\right)$ of the free part,

$$P\left(k\right)\propto {\left|{\tilde{\psi }}_{f}\left(k\right)\right|}^{2}={\left|F,\left[f\left(r\right)\;\psi \left(r;R,t_{f}\right)\right],\left(k\right),|\right.}^{2},$$ (6)

where F denotes the Fourier transform. For *t*_*f*_ > *T*, the right-hand side of Eq. 6 rapidly converges towards the final (far field) PMD (Wollenhaupt et al., 2002; Bayer et al., 2020).

## 3 Results

Motivated by recent attosecond studies on molecular FEVs (Yuan et al., 2016; Djiokap et al., 2018; Guo et al., 2021), we start in Section 3.1 by considering a rigid diatomic molecule interacting with various standard pulse shapes, including simple linearly and circularly polarized pulses as well as counterrotating circularly polarized double pulse sequences (Pengel et al., 2017b). For a selection of fixed internuclear separations, we analyze the PMD resulting from SPI and MPI to identify the basic physical mechanisms in the creation of molecular FEVs. Molecular vibration is introduced in Section 3.2, where we study the influence of the nuclear motion on the electronic excitation for SPI and MPI. In Section 3.3, we address different types of spatial averaging in view of the experimental implementation of molecular FEV scenarios. For this purpose, we consider a CEP-sensitive bichromatic two- vs. three-photon ionization scenario to compare the calculated molecular frame PMD with the PMD measured in the laboratory frame. In particular, we investigate the influence of molecular rotation averaging and CEP-averaging, both of which play a crucial role in molecular photoionization experiments.

### 3.1 Rigid Molecule

**FIGURE 2:**
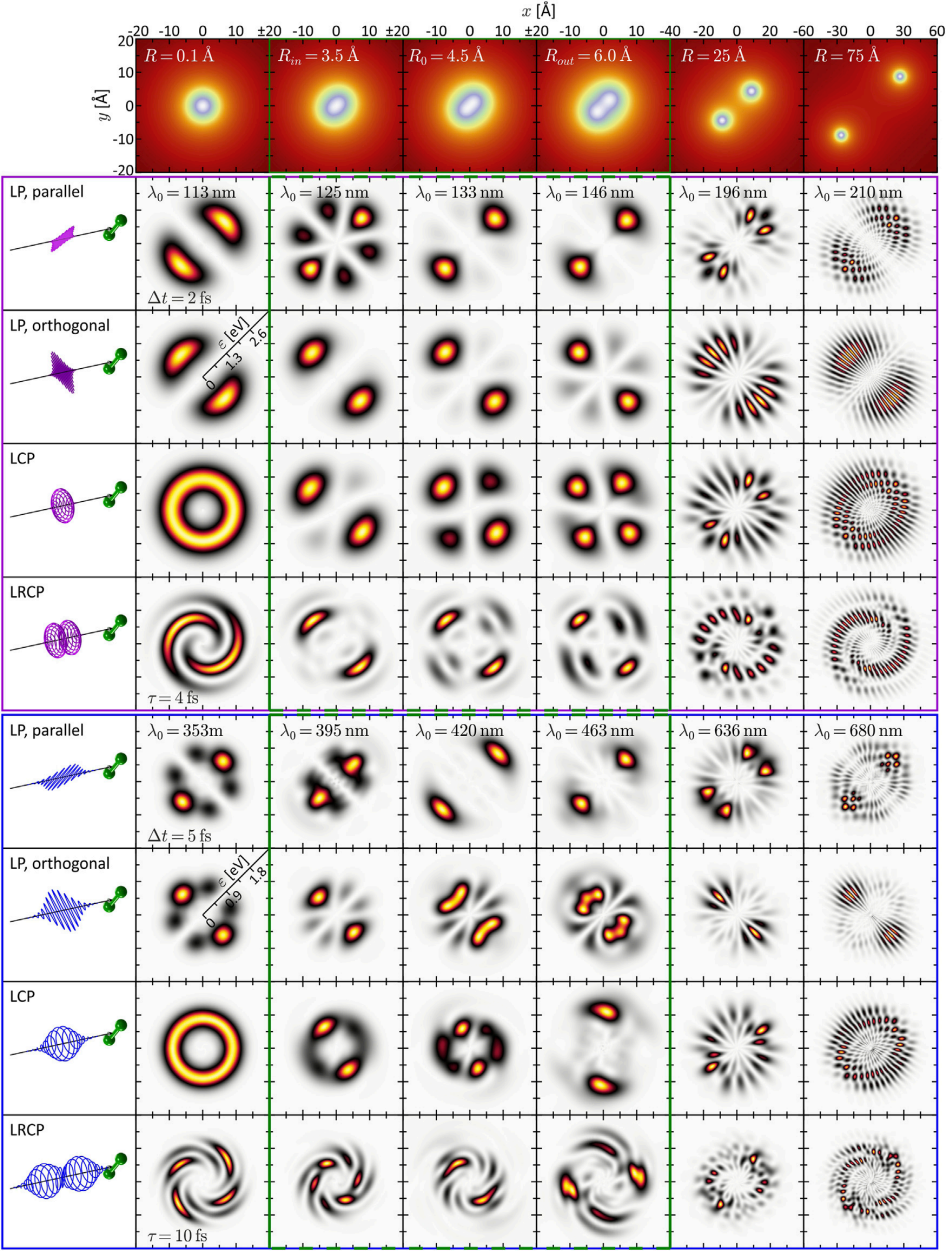
(Color online.) Gallery of calculated and energy-calibrated PMDs created by photoionization of the rigid molecule at different internuclear separations (columns) with different types of laser pulses (rows). The corresponding molecular potentials are shown in the top row. The laser pulses are illustrated on the left-hand side. The upper part (magenta frame) shows the PMDs resulting from one-photon ionization, the PMDs in the lower part (blue frame) result from three-photon ionization. The green frames (second to fourth column) indicate the vibration window.

To get a first idea of the ionization dynamics of diatomic molecules, especially when ionized with polarization shaped pulses, we start with a rigid diatomic molecule whose nuclei are fixed in space. This so-called static nuclear frame represents a particularly good approximation for the interaction of molecules with attosecond pulses (Yuan et al., 2016; Djiokap et al., 2018; Guo et al., 2021), because the nuclei are virtually frozen on the attosecond timescale. For our purpose, freezing the nuclei serves as a simplification to provide a clear physical picture of the various interference mechanisms involved in molecular photoionization. The influence of the nuclear motion is investigated next in Section 3.2. In the following, two types of perturbative ionization processes are compared. First, we study SPI by XUV/UV pulses with central wavelengths in the range of *λ*_0_ = 113−210 nm and a fixed pulse duration of Δ*t* = 2 fs. Second, we study MPI by UV/VIS pulses with central wavelengths of *λ*_0_ = 353−680 nm and a duration of Δ*t* = 5 fs. The pulse duration in the MPI scheme is chosen slightly larger than in the SPI scheme, because the photoelectron signal from (non-resonant) perturbative *N*-photon ionization is determined by the *N*-th order optical spectrum (Meshulach and Silberberg, 1999). For a Gaussian-shaped pulse, the signal is spectrally broadened by a factor of $\sqrt{N}$ . The effective pulse duration of the 5 fs pulse in the three-photon ionization scheme discussed in Sections 3.1.2, 3.2.2 is hence $\Delta t_{\mathrm{eff}}=\Delta t/\sqrt{3}=2.9\;\text{fs}$ , which is close to the pulse duration in the SPI scheme. Also, with this choice, the XUV and the VIS pulses have approximately the same number of optical cycles. In general, the results from SPI are conceptually more transparent and easier to interpret than the MPI results, because SPI is not influenced by intermediate bound state resonances and dipole selection rules for the absorption of multiple photons. On the other hand, MPI schemes are more amenable to the experimental implementation, since polarization-shaped ultrafast VIS and UV laser sources are readily available in the lab. We investigate the PMD created by photoionization of the potassium molecular ion (cf. Section 2.1) by standard types of polarization-shaped laser pulses at different internuclear separations *R*. Figure 2 shows a gallery of the numerical results in kinetic energy representation. Different rows correspond to the results obtained for different pulse shapes, including linearly polarized (LP) single pulses with their polarization vector aligned either parallel or orthogonal to the internuclear axis, left-handed circularly polarized (LCP) single pulses and double pulse sequences consisting of an LCP first pulse and a right-handed circularly polarized (RCP) second pulse (LRCP sequence) separated by *τ* = *τ*_2_ − *τ*_1_ = 2Δ*t*. The results obtained for different *R* are organized in columns, with the related molecular potentials V(r;R) shown in the top frames. Three distinct *R*-regions are selected. The first column corresponds to the atomic limit, *R* ≪ *R*_0_, where the potential resembles that of a single atom. The second to fourth column (green frame) corresponds to the vibration window *R*_*in*_ ≤ *R* ≤ *R*_*out*_ discussed in the following sections, including the equilibrium distance *R* = *R*_0_ (third column). The fifth column represents the transition to the dissociation limit, *R* ≫ *R*_0_, which is covered in the last column. Because the ionization potential depends on *R*, the central wavelength *λ*_0_ is adapted in each column such that the PMD is centered in the same kinetic energy window, i.e., [0, 2.6] eV in the SPI scheme (magenta frame) and [0, 1.8] eV in the MPI scheme (blue frame).

#### 3.1.1 Static One-Photon Ionization

We start by inspecting the results from the SPI scheme. In the limit *R* → 0 (first column of Figure 2), the PMDs are straightforwardly described in an atomic picture. The potential becomes approximately spherically symmetric entailing an |*s*, *m* = 0⟩-type ground state wave function. In accordance with the dipole selection rules, SPI by an LP laser pulse therefore creates a |*p*, 0⟩-type, dumbbell-shaped photoelectron wave packet aligned along the polarization direction. Similarly, an LCP pulse produces a |*p*, 1⟩-type, torus-shaped wave packet. In general, the superposition principle applies to perturbative SPI, i.e., the free electron wave packet from photoionization with a superposition of laser fields is given by the coherent superposition of the partial waves from the individual fields (Wollenhaupt et al., 2002). For example, the photoelectron wave packets created by an LCP pulse are a coherent superposition of the wave packets created by the corresponding LP pulses parallel and orthogonal to the internuclear axis. SPI by a time-delayed LRCP sequence yields an FEV in the shape of a two-armed Archimedean spiral with a counterclockwise sense of rotation (Ngoko Djiokap et al., 2015). This spiral-shaped PMD is understood in the framework of the perturbative ionization model presented in Pengel et al. (2017a), Pengel et al. (2017b) applied to one-photon ionization. According to the model, the two-armed FEV arises from the superposition of the ${\tilde{\psi }}_{p,1}\left(k\right)$ - and ${\tilde{\psi }}_{p,-1}\left(k\right)$ -type partial waves created by the LCP and RCP pulse, respectively. Due to the free time evolution during the time *τ* between both pulses, the initial wave packet acquires an energy-dependent phase of −*ɛ*(*k*)*τ*/*ℏ*, where *ɛ*(*k*) = *ℏ* ^2^ *k* ^2^/(2*m*_*e*_) is the photoelectron kinetic energy. The PMD of the two-armed FEV thus reads $P\left(k\right)=|{\tilde{\psi }}_{p,1}\left(k\right)e^{-i\varepsilon \left(k\right)\tau /\hbar }+{\tilde{\psi }}_{p,-1}\left(k\right){|}^{2}$ . The number of spiral arms is determined by the difference between the magnetic quantum numbers of the involved partial waves. The rotational sense depends on the pulse ordering and is inverted for an RLCP sequence.

**FIGURE 3:**
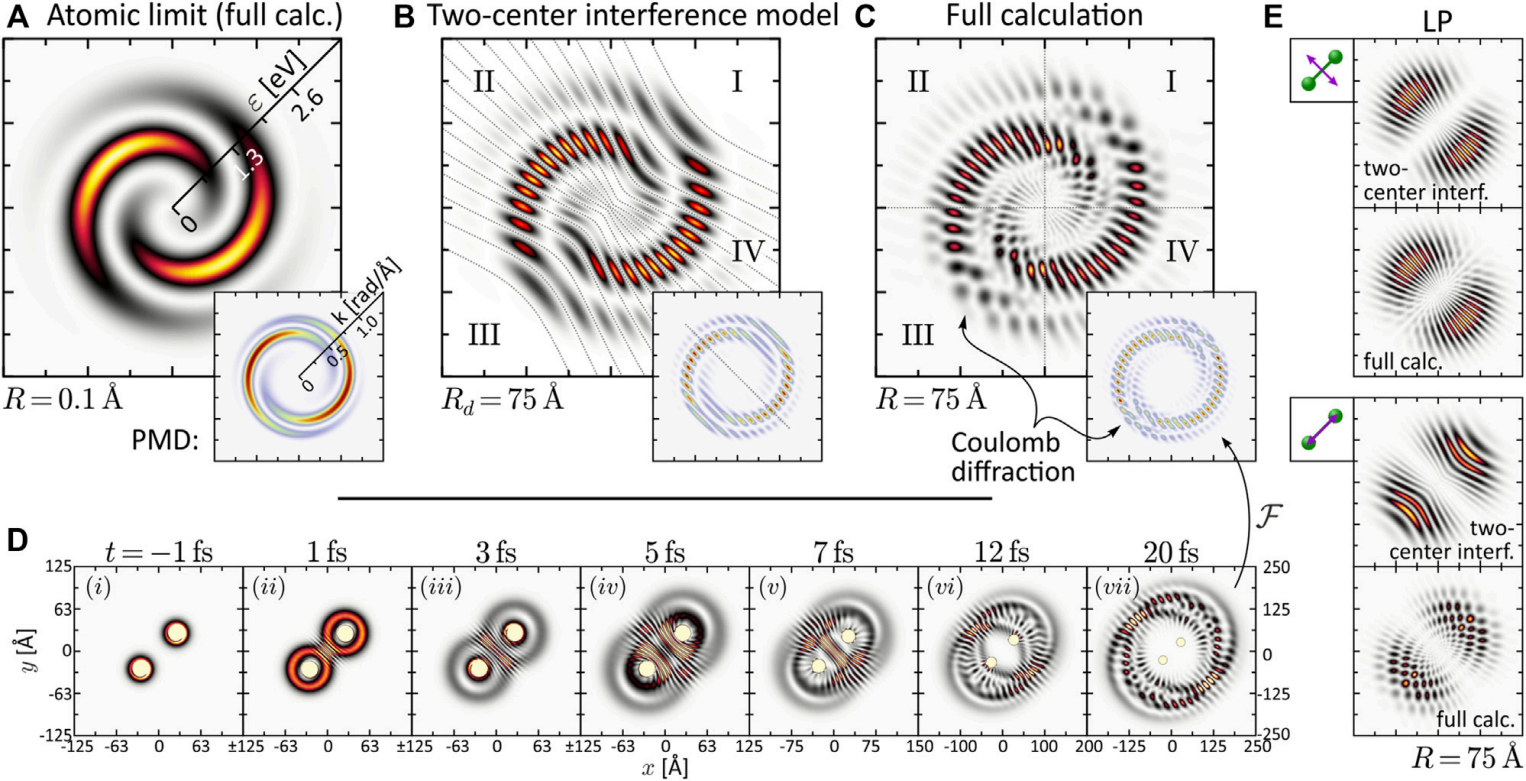
(Color online.) Discussion of interference mechanisms on the example of the two-armed FEV created by one-photon ionization with an LRCP pulse sequence (cf. Panel 2). The two pulses are centered at *τ*_1_ = −*τ*_2_ = −2 fs. **(A)** Energy-calibrated PMD of the FEV in the atomic limit *R* = 0.1 Å. The inset shows the actual PMD in momentum representation. **(B)** Two-center interference model based on the superposition of two atomic partial wave packets with origins displaced by *R*_*d*_ = 75 Å. **(C)** Full calculation of the two-armed FEV in the dissociation limit *R* = 75 Å. **(D)** Time series of the electron density |*ψ*(***r***; ***R***, *t*)|^2^ illustrating the diffraction of the partial wave packets emitted from one nucleus at the Coulomb potential of the neighboring nucleus. **(E)** Comparison between two-center interference model and full calculation for the LP single-pulse scenarios orthogonal and parallel to the internuclear axis.

Next, we consider the PMD from SPI in the dissociation limit *R* ≫ *R*_0_. The PMDs shown in the last column of Figure 2 exhibit the same coarse (average) structure as in the atomic limit but are modulated by an intricate interference pattern. Some of the PMDs resemble the trilobite states currently discussed in the context of long-range Rydberg molecules (Shaffer et al., 2018; Eiles, 2019) and initially reported in (Greene et al., 2000). The observed pattern results from the interplay between two interference mechanisms already discussed by Cohen and Fano (1966) and reviewed recently in Yuan and Bandrauk (2015). The first mechanism is two-center interference, i.e., the superposition of partial waves emitted from the individual nuclei—hence both being of similar shape as in the atomic limit—spatially displaced by *R*. The second mechanism is Coulomb diffraction of each partial wave at the potential of the neighboring nucleus. Both mechanisms are illustrated in Figure 3 on the example of the two-armed FEV from ionization with an LRCP sequence. Panel (a) shows the spiral-shaped PMD $|\tilde{\psi }\left(k\right){|}^{2}$ in the atomic limit, *R* = 0.1 Å, in energy and momentum representation (inset). Panel (b) shows the result of a simple two-center interference model based on the superposition of two replica of the atomic wave function from (a) with spatial origins displaced by *R*_*d*_ = 75 Å. According to the Fourier shift theorem, the spatial displacement gives rise to a relative linear phase in momentum space − ***R***_*d*_ ⋅ ***k*** between the two partial waves in the direction of the internuclear axis. The resulting PMD $|\tilde{\psi }\left(k\right)+\tilde{\psi }\left(k\right)e^{-iR_{d}\cdot k}{|}^{2}=2|\tilde{\psi }\left(k\right){|}^{2}\left[1+\cos\left(R_{d}\cdot k\right)\right]$ , shown in the inset of (b), exhibits a cosine modulation along the positive grid-diagonal. The nodal lines are aligned parallel to the negative diagonal (grey dotted line in the inset). The energy calibration *k*↦*ɛ*(*k*) of the PMD bends the linear nodal lines and focuses them towards the center, as indicated by the grey dotted curves in the energy representation. The full calculation result for *R* = 75 Å is shown in panel (c). In the second (II) and fourth (IV) quadrant of the grid, i.e., lateral to the internuclear axis, the fringe-type interference pattern indeed resembles that of the two-center interference model. However, in axial direction, i.e., in the first (I) and third (III) quadrant, the full calculation exhibits a speckle-type interference pattern. This speckle pattern is a superposition of the two-center interference pattern emanating laterally from the internuclear axis, and a second pattern emanating in axial direction. The latter arises from the diffraction of the partial waves emitted from either nucleus at the Coulomb potential of the neighboring nucleus. Panel (d) illustrates the Coulomb diffraction process by a time series of the coordinate space density |*ψ*(***r***; ***R***, *t*)|^2^ (note the different grid ranges). The series begins with the creation of the |*p*, 1⟩-type partial waves by the initial LCP pulse centered at *τ*_1_ = −2 fs (frame (*i*)). Both partial waves evolve rapidly into torus shapes which overlap and interfere in between the two nuclei (frame (*ii*)). By the time the |*p*, − 1⟩-type partial waves are created by the time-delayed RCP pulse (frame (*iii*)), the initial waves have already arrived at the neighboring nuclei. Frames (*iii*)–(*v*) capture their diffraction at the attractive Coulomb potentials which focus them towards the internuclear axis. The time-delayed partial waves undergo the same diffraction process in frame (*vi*). Eventually, all partial waves have passed the two nuclei and depart from the molecule [frame (*vii*)], dispersing asymptotically into their own PMD (Winter et al., 2006; Bayer et al., 2020). To highlight the different contributions of two-center interference and Coulomb diffraction to the PMD, panel (e) compares full calculation and two-center interference model for the LP scenarios parallel and orthogonal to the internuclear axis (see first and second frame of last column in Figure 2). In the orthogonal case (top), where the PMD is aligned lateral to the molecular axis, the agreement of the model and calculation is quite convincing. In the parallel case (bottom), however, where the PMD is aligned along the molecular axis, the deviations are substantial, emphasizing the strong influence of Coulomb diffraction on the interference pattern in this direction. In summary, besides Coulomb diffraction, the seemingly complicated molecular FEV in Figure 3C can be understood as a result an interplay of three types of interference: (1) The interference of the two atomic wave packets ${\tilde{\psi }}_{p,\pm 1}\;\propto e^{\pm im\phi }$ , created by the two counterrotating circularly polarized subpulses, determines the azimuthal structure of the PMD, (2) by virtue of the time delay *τ*, the two pulses act as a *temporal double slit* due to the free time-evolution phase factor *e* ^−*iɛτ*/*ℏ*^, which results in the radial fringe pattern in the PMD (Wollenhaupt et al., 2002), and (3) the spatial displacement ***R*** of the two atoms along the molecular axis, associated with the phase factor *e* ^−*i* ***k*** ⋅ ***R***^, causes a lateral fringe pattern in the PMD, reminiscent of a *spatial double slit*. We note, that this physical picture allows us to extract the internuclear separation directly from the lateral fringe pattern in the PMD.

While the interpretation of the PMDs in the atomic and the dissociation limit is quite transparent, the analysis of the PMDs in the intermediate regime, presented in the central columns of Figure 2, is more demanding. The penultimate column, with *R* = 25 Å, represents the transition between the two limits. Here the PMDs are already characterized by the two interference mechanisms, the main difference being the lower modulation frequency of the two-center interference fringes in comparison to the last column. Around the equilibrium distance *R*_0_ = 4.48 Å (third column; see also Figure 1A) of the molecule, however, the two mechanisms are less meaningful for the interpretation of the PMDs. In this regime, the internuclear separation is difficult to extract directly from the PMD but could be retrieved by comparison to an analytical model of the photoelectron wave function (Fernández et al., 2009) or by application of a machine learning procedure (Shvetsov-Shilovski and Lein, 2022). Some general features of the PMDs, which we confirmed numerically, can be identified. For example, the PMDs created by LCP and RCP pulses (the latter are not shown) are mirror images of one another, when reflected at the internuclear axis or orthogonal to this axis. Mirroring the field at one of these axes inverts its rotational sense and transforms an LCP into an RCP pulse and vice versa. Currently, the mirror symmetry of LCP and RCP pulses is also referred to when discussing chiro-optical effects, such as photoelectron circular dichroism (Hergenhahn et al., 2004; Powis, 2008; Lux et al., 2012). Altogether, the second (*R* = 3.45 Å), third and fourth (*R* = 6.0 Å) column correspond to the nuclear vibration window which will be investigated in the following Section 3.2. The PMDs shown here build the basis for the discussion of the vibrational dynamics.

#### 3.1.2 Static Three-Photon Ionization

Unlike SPI, MPI depends not only on the initial state and the final state in the continuum, but is also highly sensitive to intermediate resonances. Moreover, the absorption of multiple photons opens up numerous ionization pathways leading to target continuum states characterized by superpositions of angular momenta that are generally larger than in SPI. As a consequence, PMDs created by MPI have a richer structure than those created by SPI. The simulation results shown in the bottom part of Figure 2 (blue frame) are based on three-photon ionization of the potassium molecular ion, motivated by recent experimental studies on potassium atoms (Wollenhaupt et al., 2009a; Pengel et al., 2017a; Pengel et al., 2017b) and molecules (Bayer et al., 2013; Braun et al., 2014). The PMDs in the first column are again well understood in an atomic picture invoking the dipole selection rules. Accordingly, three-photon ionization of the |*s*, 0⟩-type ground state by an LP pulse creates an |*f*, 0⟩-type photoelectron wave packet aligned in laser polarization direction (Wollenhaupt et al., 2009a). Analogously, the LCP pulse creates an |*f*, 3⟩-type, torus-shaped wave packet, slightly more confined in radial direction compared to the SPI scheme. The radial width of the PMD in the MPI scheme is determined by the bandwidth of the third-order spectrum of the 5 fs pulse, which is actually smaller than the fundamental bandwidth of the 2 fs pulse. The LRCP sequence creates a six-armed FEV with counterclockwise sense of rotation, as observed and discussed in detail in Pengel et al. (2017a), Pengel et al. (2017b).

In the dissociation limit (last column), we recognize the same interference mechanisms at play as in the SPI scheme (cf. Section 3.1.1). Again, the average structure of the PMDs is the same as in the atomic limit (first column). Along the internuclear axis, however, the PMDs are modulated by speckle-shaped interference patterns due to the combined effect of Coulomb diffraction and two-center interference, whereas in lateral direction, we observe the fringe-type patterns characteristic for almost pure two-center interference.

The interpretation of the intermediate *R*-regime is more involved. The PMDs shown in the central columns depend strongly on the central wavelength *λ*_0_, which indicates the presence of intermediate resonances. Also, a comparison of the different PMDs within each row shows that the shape of the PMDs changes drastically with the internuclear separation. In contrast, in the SPI scheme, where resonances of the bound system play no role, the PMDs in the vibration window (green frame) are qualitatively similar and change smoothly with *R*. Another indication of intermediate resonances is the rotation of the PMDs in the circularly polarized scenarios, discernible e.g., by the angular alignment of the main lobes in the 2D plane. Resonances introduce additional ionization phases in the photoelectron wave packet (Eickhoff et al., 2022) which, in the circularly polarized case, translate into an azimuthal rotation of the PMD (Eickhoff et al., 2020a). The observed rotation is particularly pronounced in the PMDs from LCP and LRCP ionization in the fourth column, i.e., for *R* = *R*_*out*_ = 6.0 Å. The excitation scheme in Figure 1A suggests that at this internuclear separation the transition from the ground state to state *ψ*_6_ (green line in Figure 1A) becomes near-resonant with a pulse of central wavelength *λ*_0_ = 463 nm (see also Figure 6B).

**FIGURE 4:**
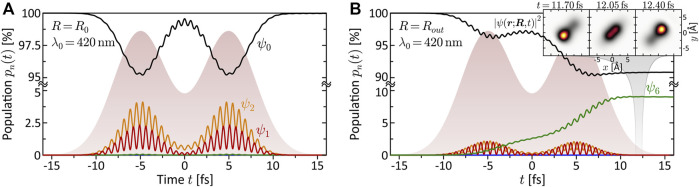
(Color online.) Bound state population dynamics of the rigid molecule excited by the LRCP sequences (shaded background) from the MPI scheme. Shown are the time-dependent populations of the eight lowest electronic states *ψ*_*n*_ (cf. Panel 1B). **(A)** Non-resonant excitation at the equilibrium distance *R*_0_ = 4.48 Å using a central wavelength of *λ*_0_ = 420 nm. Only states *ψ*_1_ and *ψ*_2_ are transiently populated, mediating the MPI process. Finally, almost the entire population returns to the ground state. **(B)** Resonant excitation of state *ψ*_6_ at the outer turning point *R*_*out*_ = 6.0 Å, using the same central wavelength *λ*_0_ = 420 nm as in **(A)**. The coherent superposition of states *ψ*_0_ and *ψ*_6_ corresponds to an efficient ultrafast charge oscillation in the bound molecular system, illustrated in the inset by a time series of the electron density |*ψ*(***r***; ***R***, *t*)|^2^.

The quantitative analysis of the bound state population dynamics *p*_*n*_(*t*) for the lowest eight electronic states is shown in Figure 4. At the equilibrium distance *R*_0_ and *λ*_0_ = 420 nm (third column), shown in panel (a), we see the transient population of states *ψ*_1_ and *ψ*_2_ by a few percent, followed by a population return to the ground state. This result confirms the non-resonant character of the interaction and verifies the perturbative interaction conditions. Panel (b) shows the dynamics at *R*_*out*_ and the same central wavelength. In this case, we observe an efficient population transfer to state *ψ*_6_ of about 10%. The induced electronic coherence results in a pronounced charge oscillation in the bound molecular system along the internuclear axis. This dynamics is illustrated in the insets to (b) which show the time-dependent electron density |*ψ*(***r***; ***R***, *t*)|^2^ over one half-cycle of the charge oscillation after the interaction. These findings confirm the above assumption of a resonance-enhanced multiphoton ionization (REMPI) process at *R*_*out*_ *via* state *ψ*_6_. Our simulations show that the exact resonance at *R*_*out*_ is found around *λ*_0_ = 413 nm. At *λ*_0_ = 463 nm, as in the fourth column, we still register about 1% population transfer due to the broad bandwidth of the pulses. Additional high-lying resonances are expected at the two-photon level, due to the high density of Rydberg states, but their analysis is beyond the scope of this paper. The resonance *ψ*_0_ → *ψ*_6_ at *R*_*out*_, however, will also be relevant in the discussion of coupled vibrational and electronic (vibronic) MPI dynamics in Sections 3.2.2, 3.3.

### 3.2 Vibrating Molecule

The coupled electron-nuclear dynamics in molecules has been studied extensively on the femtosecond timescale, as reviewed for example in Gatti (2014), Bircher et al. (2017), and very recently on the attosecond timescale (Nisoli et al., 2017; Cattaneo et al., 2018). Experimentally, the coupled electron-nuclear dynamics of potassium dimers has been investigated in Bayer et al. (2013), Braun et al. (2014). In this section, we account for the vibration of the nuclei along the internuclear axis and study the coupling between the nuclear motion and the electronic ionization dynamics. To this end, we initialize the molecule in a vibrationally excited state of the electronic ground state potential *V*_0_(*R*), which we can think of as being prepared, e.g., by stimulated Raman excitation. We use LRCP sequences to study the creation of FEVs by ionization of the vibrating molecule. Femtosecond double pulse sequences are well-established in femtochemistry and the time-resolved investigation of molecular dynamics (Zewail, 1995), being the basis of pump-probe techniques (Zewail, 2000). Here, however, the focus is not primarily on time-resolved imaging of the dynamics, but on exploring their signatures imprinted in the photoelectron spirals. In the following, we design specific vibration-scenarios *via* the timing of the pulse sequence. Specifically, we lock the two pulses to distinct stages of the nuclear oscillation, i.e., the inner and the outer turning point. The resulting PMD is analyzed for both the SPI and the MPI scheme (cf. Section 3.1).

#### 3.2.1 Dynamic One-Photon Ionization

The timescale of the vibrational dynamics depends, amongst others, on the reduced mass *μ* of the nuclear system. For the purpose of this study, *μ* is chosen such that the nuclear timescale is adapted to the laser pulses employed in Section 3.1.1, which yields the most transparent results. Specifically, *μ* is adapted such that the period of the vibration (cf. top inset to Figure 1) is *T*_*v*_ = 13.7 fs. This value is compatible with the laser pulse duration of Δ*t* = 2 fs. The laser central wavelength is fixed to *λ*_0_ = 125 nm as in the second column of Figure 2 (magenta frame). The variation parameter is the time-delay *τ* between the two subpulses of the LRCP sequence.

**FIGURE 5:**
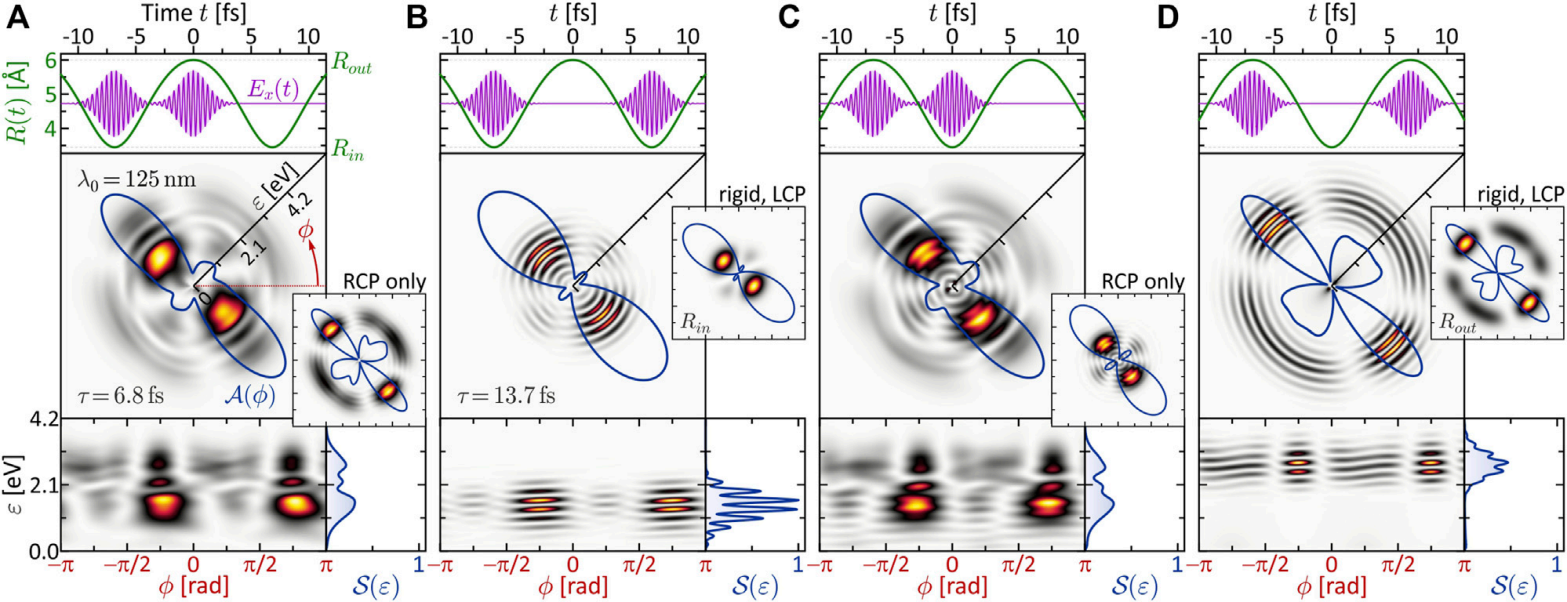
One-photon ionization of the vibrating molecule by single-color LRCP sequences locked to the two turning points of the nuclear vibration *R*(*t*). The 2D PMD in energy representation P(ε,ϕ) is shown in Cartesian (central frames) and spherical representation (bottom frames). In addition, the energy-integrated angular distributions A(ϕ) are shown as polar plots (blue curves) and the angle-integrated energy spectra S(ε) are shown in the bottom right frames. In **(A,C)**, the two subpulses are locked to different turning points, while in **(B)**, both pulses ionize the molecule at the inner and in **(D)** at the outer turning point.

**FIGURE 6:**
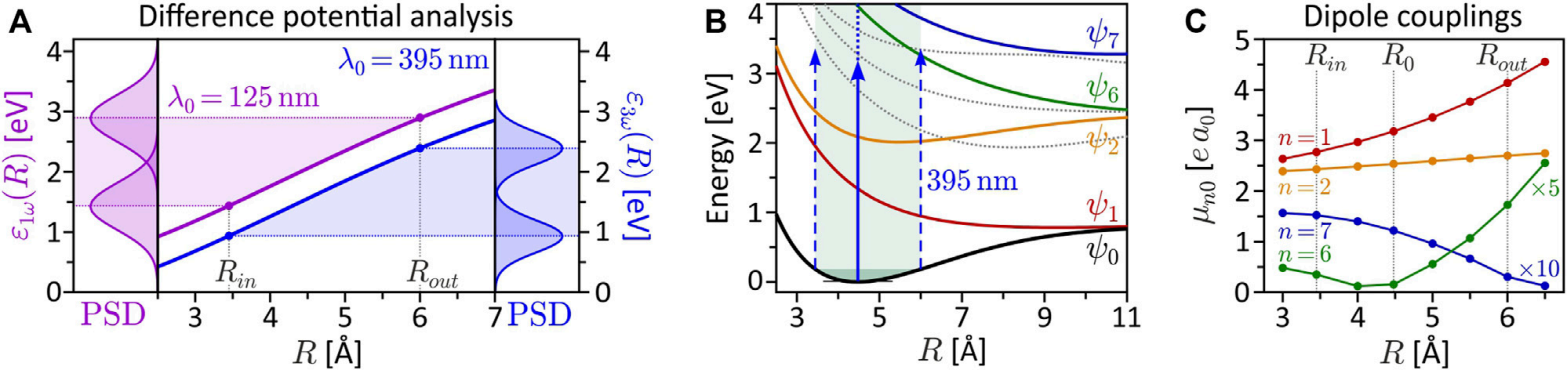
(Color online.) Derived quantities to characterize the interdependence of electronic and nuclear dynamics. **(A)** Difference potential analysis for the SPI scheme (magenta curve) and the MPI scheme (blue curve). The left frame depicts the power spectral density (PSD) of the 125 nm, 2 fs pulse mapped at the inner and outer turning point of the vibration. The right frame shows the PSD of the third order spectrum of the 395 nm, 5 fs pulse. **(B)** Excitation scheme of the vibrating molecule interacting with the 395 nm pulse employed in the MPI scheme. At the outer turning point, state *ψ*_6_ is excited resonantly from the ground state. **(C)** *R*-dependent transition dipole moments *μ*_*n*0_(*R*) = |*e*⟨*ψ*_*n*_| ***r*** |*ψ*_0_⟩| for ground state transitions to the seven lowest bound electronic states (vanishing dipole couplings are not shown). At the outer turning point *R*_*out*_, the coupling of the ground state to the resonant state *ψ*_6_ is one order of magnitude larger than at the equilibrium distance *R*_0_.

Two distinct scenarios are investigated. First, the pulses are separated by half a vibrational cycle, *τ* = *T*_*v*_/2 = 6.84 fs, and locked to *different* turning points. Second, the pulses are separated by *τ* = *T*_*v*_ and locked to the *same* turning point of two consecutive vibration cycles. The numerical results are presented in Figure 5. The top frames show the nuclear motion *R*(*t*) (green curve) together with the *x*-component of the circularly polarized laser field ***E*** (*t*) (magenta curve). During the most intense part of the pulses, i.e. within Δ*t*, the nuclei move by about 0.2 Å at the inner and 0.1 Å at the outer turning point. The calculated photoelectron spectra are shown below in different representations, including the 2D PMD P(ε,ϕ) in a Cartesian and a spherical coordinate frame, the energy-integrated angular distribution A(ϕ) (blue polar plot) and the angle-integrated energy spectrum S(ε) (bottom right frame). We notice that the photoelectron signal is spread out over a much larger energy region than in the rigid case (cf. Section 3.1.1). This spreading, which is best discernible in the energy spectra S(ε) , is particularly apparent in panels (a) and (c). In these scenarios, the ionizing field probes the nuclear dynamics over a full vibrational cycle. In the static molecular frame, the photoelectrons are created in a fixed kinetic energy window, the width of which is essentially determined by the spectral bandwidth of the laser pulses. In the dynamic case, however, the ionization maps the nuclear motion into different energy windows *via* the *R*-dependent ionization potential Δ*V*(*R*) = *V*_*i*_(*R*) − *V*_0_(*R*). According to Mulliken’s difference potential analysis (Mulliken, 1971), a photoelectron created at the internuclear separation *R* by absorption of a photon with frequency *ω*_0_ receives a kinetic energy of *ɛ*_1*ω*_(*R*) = *ℏω*_0_ − Δ*V*(*R*). Thus, the different stages of the vibrational dynamics are energetically disentangled in the energy spectrum, which is the basis for the time-resolved mapping of ultrafast vibrational wave packets by femtosecond photoelectron spectroscopy (Baumert et al., 1991; Assion et al., 1996; Wollenhaupt et al., 2003; Bayer et al., 2013; Braun et al., 2014). In a full quantum mechanical treatment of the vibrational dynamics, the photoelectron energy spectrum is additionally broadened due to the finite width of the vibrational wave packet. The latter describes a distribution of internuclear separations at which the ionization takes place. To estimate this additional broadening, the entire vibrational wave packet needs to be mapped onto the photoelectron energy axis *via* the difference potential. The result of the difference potential analysis applied to the SPI scheme is shown as magenta curve in Figure 6A. Taking into account the laser bandwidth in addition, depicted in the left frame, the kinetic energy range of the released photoelectrons is *ɛ* ∈ [0; 4.2 eV].

Specifically, the inner turning point is mapped to an energy of *ɛ*_1*ω*_(*R*_*in*_) = 1.4 eV, while the outer turning point is mapped to *ɛ*_1*ω*_(*R*_*out*_) = 2.9 eV. This mapping gives rise to the localized rings observed in Figures 5B,D. In these SPI scenarios, the laser pulses probe the nuclear dynamics at two consecutive inner and outer turning points, respectively. The enhancement of the photoelectron amplitude in these regions indicates the increased probability of finding the nuclei at one of the two turning points, in analogy to a classical oscillator. The corresponding angular distributions A(ϕ) are almost identical to the result obtained in the rigid case. For comparison, the PMDs created by SPI of the rigid molecule using a single LCP pulse is shown in the respective insets. Note that, in the single pulse scenarios, the side lobes of the angular distribution are not symmetric. This asymmetry is compensated in the LRCP scenarios by the contribution of the time-delayed RCP pulse, which is mirrored at the internuclear axis (see also discussion in Section 3.1.1). The delicate radial fringe patterns observed in the LRCP scenarios results from the interference of the two partial wave packets created by the LCP and the RCP subpulse, enabled by their energetic overlap. The fringe spacing is determined by the time-delay as Δ*ɛ* = *h*/*τ* (Wollenhaupt et al., 2002; Pengel et al., 2017b). In the polar representation of the 2D PMD (bottom left frames), we recognize that the fringes are slightly sloped, suggesting a spiral-shaped interference pattern analogous to that of atomic FEVs (Ngoko Djiokap et al,. 2015; Pengel et al., 2017b). In contrast to the atomic case, however, the spiral arms of these molecular FEVs evolve non-linearly in the polar plane. This non-linear *ϕ*-dependence of the interference pattern, which is neither fully resolved in the energy spectrum S(ε) nor visible in the angular distribution A(ϕ) , encodes spectroscopic information about the structure and the dynamics of the molecule.

In panels (a) and (c) of Figure 5, we observe no such regular interference pattern as in (b) and (d). Here, the two partial wave packets are mapped into different energy windows and interfere only in a narrow overlap region around *ɛ* = 2.1 eV (see left frame of Figure 6A), where indeed some interference fringes are observed. The energetic disentanglement of the free electron wave packets is also indicated by the slight asymmetry of the side lobes in the angular distribution A(ϕ) . These side lobes reflect mainly the side lobes of the low-energy wave packet created at the inner turning point, i.e., by the LCP subpulse in panel (a) and the RCP subpulse in panel (c). A more striking difference between the PMDs in panel (a) and (c) is the weak fringe pattern which is observed in the low-energy region *ɛ* ≲2.1 eV in (c) and absent in (a). This difference is seemingly counterintuitive, since the underlying physical scenarios are—at first glance—very similar. A closer numerical investigation revealed that the fringe pattern arises due to non-adiabatic dynamics of the electronic ground state wave function induced by the vibrating nuclei. While the ground state electron density |*ψ*(***r***; ***R***, *t*)|^2^ adapts almost adiabatically to the nuclear motion, the corresponding wave function acquires a saddle-shaped phase aligned along the internuclear axis. Signatures of this phase emerge as the radial fringe pattern in the PMD in (c). To confirm this analysis, we examined the PMD created by the time-delayed RCP pulse only, i.e., without the initial LCP subpulse. The resulting PMD, shown in the inset to (c) exhibits the same fringe pattern as the full calculation. The same procedure applied to the scenario in (a) yields the PMD shown in the inset to (a). Evidently, the wave packet created by the RCP pulse at the outer turning point also exhibits a fringe structure reflecting the vibrational history of the molecule, albeit less pronounced as in (c) and therefore less visible in the full calculation. In both cases, we find for the fringe spacing Δ*ɛ* = 334 meV. This value corresponds to a time constant of *τ* = *h*/Δ*ɛ* = 12.4 fs, approximately matching the time interval from the initiation of the vibration at *t*_*i*_ = −11.5 fs to the arrival of the RCP pulse at *t* = 0. These findings demonstrate that the interference structures in the photoelectron spectrum are highly sensitive to non-adiabatic electron-nuclear dynamics in molecules. The results once again underscore that differential detection of photoelectron wave packets is essential to reveal the subtle changes in the bound electronic wave function, which are otherwise not detected.

#### 3.2.2 Dynamic Three-Photon Ionization

**FIGURE 7:**
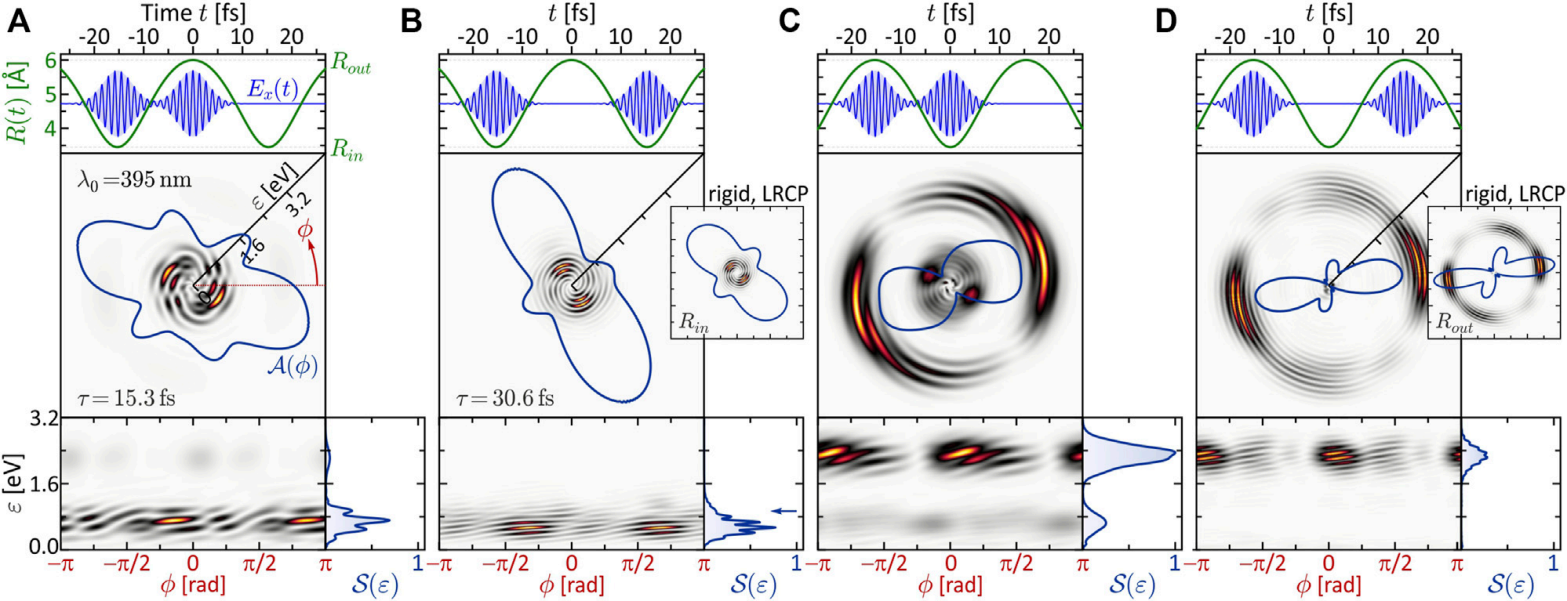
(Color online.) Three-photon ionization of the vibrating molecule by single-color LRCP sequences locked to the two turning points of the nuclear vibration *R*(*t*). The organization of panels **(A–D)** is analogous to Figure 5. Striking differences to the SPI scheme are observed, especially in panels **(A,C)**, which are attributed to the occurrence of transient intermediate resonances.

The results obtained in the dynamic MPI scheme are especially rich in information. Not only is MPI sensitive to intermediate resonances, but unlike the static case, in the presence of nuclear dynamics, electronic resonances may occur at certain internuclear distances and be absent in others. To study the signatures of such transient resonances in the PMD, we again lock two pulses, which are short compared to the vibrational period of the molecule, to different stages of the nuclear dynamics. The vibrational dynamics are adapted to the longer pulse duration of Δ*t* = 5 fs used in Section 3.1.2 by choosing the reduced mass of the nuclei such that the vibration period is increased to *T*_*v*_ = 30.6 fs. We employed the LRCP sequences from Section 3.1.2 with a central wavelength of *λ*_0_ = 395 nm (second column of Figure 2). This central wavelength is very close to the one-photon resonance *ψ*_0_ → *ψ*_6_ at the outer turning point *R*_*out*_ of the vibration, as illustrated in the excitation scheme shown in Figure 6B (see also Section 3.1.2). At the inner turning point *R*_*in*_ and at the equilibrium distance *R*_0_, however, the field is far detuned from any one-photon resonance, since the states *ψ*_3_, *ψ*_4_ and *ψ*_5_ (gray dashed curves) are not dipole-coupled to the ground state. Consequently, we expect the transient resonance *ψ*_0_ → *ψ*_6_ to play a prominent role in the scenarios involving MPI at the outer turning point. The *R*-dependent transition dipole moments plotted in Figure 6C show that the coupling between the ground state and the *ψ*_6_-state (green curve) at the outer turning point is one order of magnitude larger than at the equilibrium distance (cf. Figure 1B). In general, the variation of a transition dipole moment along a nuclear coordinate can be indicative of a change of the electronic structure in this direction (Wollenhaupt et al., 2003). However, in this case, the variation of the dipole coupling is attributed to the *R*-dependent contributions of the inner and outer lobes of *ψ*_6_ to the matrix element (cf. Figure 1B). In the vicinity of *R*_0_, at *R* ≈ 4.2 Å, these contributions cancel each other and the matrix element vanishes. The numerical results are presented in Figure 7, with the same organization as in Figure 5 to facilitate the comparison. We start with the discussion of panel (b), where both pulses ionize the vibrating molecule at the inner turning point. This is the only scenario, where the above-mentioned resonance is not crucial. According to the difference potential analysis in Figure 6A (blue curve), the inner turning point is mapped to an energy of *ɛ*_3*ω*_(*R*_*in*_) = 3*ℏω*_0_ − Δ*V* (*R*_*in*_) = 0.9 eV. This energy is marked by a blue arrow in the bottom right frame of Figure 7B. In fact, the calculated PMD is red-shifted towards a kinetic energy of *ɛ* = 0.6 eV, best seen in the energy spectrum S(ε) . An examination of the bound state population dynamics (not shown) confirmed that the excitation is fully perturbative, ruling out the AC Stark effect to explain the observed energy shift, and non-resonant at the one-photon level. The angular distribution A(ϕ) is similar but not identical to that of the static LRCP scenario shown in the inset. The slight rotation between both, indicating the acquisition of an additional phase in the REMPI process (Eickhoff et al., 2022), hints towards the influence of another resonance encountered during the vibration on the two-photon level. The analysis of the population dynamics yields that the high-lying state *ψ*_11_, plotted as cyan dashed-dotted line in Figure 1A, 1is excited near-resonantly by both subpulses—albeit with a population transfer of less than 1%. However, the population stored in *ψ*_11_ by the first subpulse is efficiently mapped by one photon of the second subpulse into an energy window around *ɛ* = 0.5 eV, consistent with the observed energy shift. A closer inspection of the 2D PMD reveals an intricate interference pattern. The pattern starts out as a six-arm spiral, as expected for three-photon ionization with LRCP pulses (Pengel et al., 2017b). With increasing energy, however, the pattern becomes more and more intertwined, indicating a beating of the six-armed FEV with a another contribution. Because this beating pattern is not observed in the rigid case (see inset), we conclude that the second contribution arises from an interplay of the non-adiabatic (but near-diabatic) dynamics of the ground state wave function (see discussion in Section 3.2.1) and the time-delayed mapping of the two-photon resonance *ψ*_11_ by the second subpulse.

A similar beating pattern is observed in Figure 7D, where both pulses ionize the molecule at the outer turning point. The created photoelectron wave packets are localized energetically around *ɛ*_3*ω*_(*R*_*out*_) = 2.4 eV, in accordance with the difference potential analysis in Figure 6A. Both pulses excite the *ψ*_6_-state near-resonantly driving a (1 + 2) REMPI process, which we verified numerically by evaluation of the population dynamics (not shown) similar to Figure 4. Our results show that the first subpulse transfers about 5% of population from the ground state to the resonant *ψ*_6_-state. Hence, the second pulse finds the molecule in a coherent superposition of states *ψ*_0_ and *ψ*_6_ and maps both into the continuum, the former by three- and the latter by two-photon ionization. One might suspect that the additional photoelectron contribution from state *ψ*_6_ is responsible for the observed beating pattern in the PMD. However, since the beating—unlike the *ψ*_0_ → *ψ*_6_ resonance—is absent in the static LRCP scenario shown in the inset, we conclude that the beating is indeed a signature of the non-adiabatic ground state dynamics. The same alignment of the angular distributions in the static and the dynamic LRCP scenario confirms the absence of additional resonances in the course of the nuclear vibration.

In the scenarios of panels (a) and (c), we expect again a broader energy distribution of the photoelectrons, because the laser field ionizes the molecule at both turning points. In fact, the PMD in panel (c) displays a bimodal energy spectrum S(ε) with significant contributions around *ɛ*_3*ω*_(*R*_*in*_) and *ɛ*_3*ω*_(*R*_*out*_). The first subpulse is locked to the outer turning point and hence induces (1 + 2) REMPI *via* the *ψ*_6_-state. The created photoelectron wave packet is centered energetically in the high-energy window around 2.4 eV. The second subpulse, locked to the inner turning point, creates a wave packet in the low-energy window around *ɛ* = 0.7 eV by direct (2 + 1) REMPI from the ground state *via* state *ψ*_11_. Again, the subtle radial fringe pattern superimposed on this contribution reveals the non-adiabatic phase dynamics of the bound state wave function. Due to our classical implementation of the nuclear dynamics, the high-energy contribution exhibits an additional spiral pattern. This pattern reflects the interference of the initial wave packet, created by the first subpulse directly, with another contribution created by time-delayed two-photon ionization of the *ψ*_6_-state by the second subpulse. However, in a quantum mechanical description of the vibrational dynamics, the *ψ*_6_-population excited by the first subpulse would follow the repulsive BO potential *V*_6_(*R*). Here, the second subpulse maps the *ψ*_6_-state coincidentally into the high-energy window around *ɛ* = 2.4 eV, such that the created wave packet overlaps with the first wave packet. This observation shows that, in order to account for the full dynamics of multiple vibrational wave packets in electronic superposition states, our numerical model needs to be refined towards a full quantum mechanical treatment of the coupled electron-nuclear dynamics.

Eventually, in panel (a), the role of the two subpulses is exchanged. The first subpulse ionizes the molecule at the inner turning point *via* direct (2 + 1) REMPI, creating a low-energy wave packet around *ɛ* = 0.9 eV and transferring some population to *ψ*_11_. At the outer turning point, the second subpulse therefore ionizes the molecule by direct (1 + 2) REMPI *via* the resonant *ψ*_6_-state, creating a high-energy wave packet around *ɛ* = 2.4 eV and, in addition, maps the *ψ*_11_-state into the continuum, creating another wave packet centered around *ɛ* = 0.5 eV. The interference of the two low-energy wave packets gives rise to the pronounced vortex structure observed in the low-energy region. We note that, although the different intensities of slow and fast electrons in Figures 7A,C suggest a ground state depletion by the first pulse of each scenario, the ionization is indeed perturbative. In both cases, the first pulse depopulates the ground state only by several %, comparable to Figure 4B.

The results presented in this section demonstrate that the coupled electron-nuclear dynamics leaves distinct fingerprints in the spiral-shaped PMDs of molecular FEVs. For example, the striking difference between Figures 7A,C, as well as the comparison between the PMD from the vibrating and the rigid molecule, clearly reveal that, in molecular MPI, the interplay between nuclear dynamics and electronic resonances is of great importance. Therefore, model calculations that predict the fully differential PMDs are invaluable tools to decipher the sophisticated structures of the PMD and identify the multiple interference phenomena underlying the creation of molecular FEVs. However, the results also show, that more refined models including the quantum mechanical description of nuclear dynamics are needed to accurately predict the fine details of the coupled electron-nuclear dynamics. Given the high degree of complexity of the interference patterns, further processing and analysis of the PMDs will in practice be necessary to extract detailed information on the underlying molecular dynamics. Experimentally, the creation and differential detection of molecular FEVs promises to be a highly-sensitive spectroscopic technique to probe molecular structure and dynamics, provided the delicate interference patterns are not averaged out by the experimental conditions—an issue which we will address in the following section.

### 3.3 Molecular Rotation and CEP-Averaging

So far, we have considered the laser-molecule interaction in the molecular frame, i.e., for a fixed alignment of the molecule in space. In addition, we have assumed a phase-stable wave form and polarization profile of the laser field with a fixed orientation relative to the molecular axis in the experiment. Any deviation from these idealizations will, in general, wash out the intricate interference patterns observed in the molecular frame PMD. For example, measurements are typically performed on isotropic ensembles of molecules. The PMD measured in the laboratory frame is then a rotational average of all molecular alignments relative to the laser propagation direction. In addition, the measured PMD is typically accumulated over multiple laser shots and locations within the laser focus introducing wave form averaging *via* phase fluctuations of the laser and the Gouy phase (Hoff et al., 2017; Bayer et al., 2020). While these phase fluctuations might be less significant in the case of a single multi-cycle pulse, they become crucial in the case of few-cycle pulses, multi-pulse sequences and combinations thereof. Specifically, a variation of the relative phase between the subpulses in the LRCP sequences used here for the creation of molecular FEVs rotates the spiral-shaped interference pattern in the molecular frame PMD (Pengel et al., 2017a). As a result, fluctuating relative phases blur the interference pattern in the angular direction. Since bichromatic LRCP sequences, consisting of two subpulses with commensurable central frequencies, control the rotation of the PMD *via* the relative phase and the CEP (Kerbstadt et al., 2019b; Eickhoff et al., 2020a), even CEP fluctuations wash out the observable interference pattern in the bichromatic case.

In this section, we investigate the influence of the different averaging mechanisms on the PMD in a single test scenario. Besides the averaging over the molecular alignment, we examine CEP-averaging. Unlike the relative optical phase, which can be controlled experimentally with zeptosecond precision (Köhler et al., 2011), the CEP is generally more difficult to stabilize and inherently varies over the laser focus due to the Gouy-phase (Hoff et al., 2017). Under perturbative conditions, however, FEVs created by *single-color* pulses are CEP-insensitive (Pengel et al., 2017b). Therefore, we consider a molecular FEV created by dynamic two- vs. three-photon ionization using a bichromatic (2*ω*: 3*ω*) LRCP pulse sequence, which is highly sensitive to the CEP (Kerbstadt et al., 2019b; Eickhoff et al., 2021c). The pulses are separated by a full vibrational cycle, *τ* = *T*_*v*_, and locked to consecutive instants of the nuclei traversing the equilibrium distance *R*_0_, heading outwards. Thus, both pulses probe the same vibrational stage and a maximum range of internuclear separations. Maintaining a pulse duration of Δ*t* = 5 fs as in Sections 3.1.2, 3.2.2, we adapted the reduced mass such that the vibrational period is *T*_*v*_ = 18.0 fs to fully separate both pulses in time and minimize photoelectron contributions due to frequency mixing between the two colors (Kerbstadt et al., 2017a; Eickhoff et al., 2021c). The initial LCP subpulse has a central wavelength of *λ*_1_ = 395 nm as in the MPI scheme discussed in the previous sections. The time-delayed RCP subpulse has a central wavelength of *λ*_2_ = 2*λ*_1_/3 = 263 nm to ensure the energetic overlap of two- and three-photon ionization pathways at 2*ℏω*_2_ = 3*ℏω*_1_. The amplitude ratio of the two subpulses is chosen such that in a static MPI scenario with *R* = *R*_0_ both produce the same photoelectron yield. This procedure, which is also followed in the experiments (Kerbstadt et al., 2019b; Eickhoff et al., 2020a), generally optimizes the interference contrast between the two partial wave packets in the full LRCP scenario.

**FIGURE 8:**
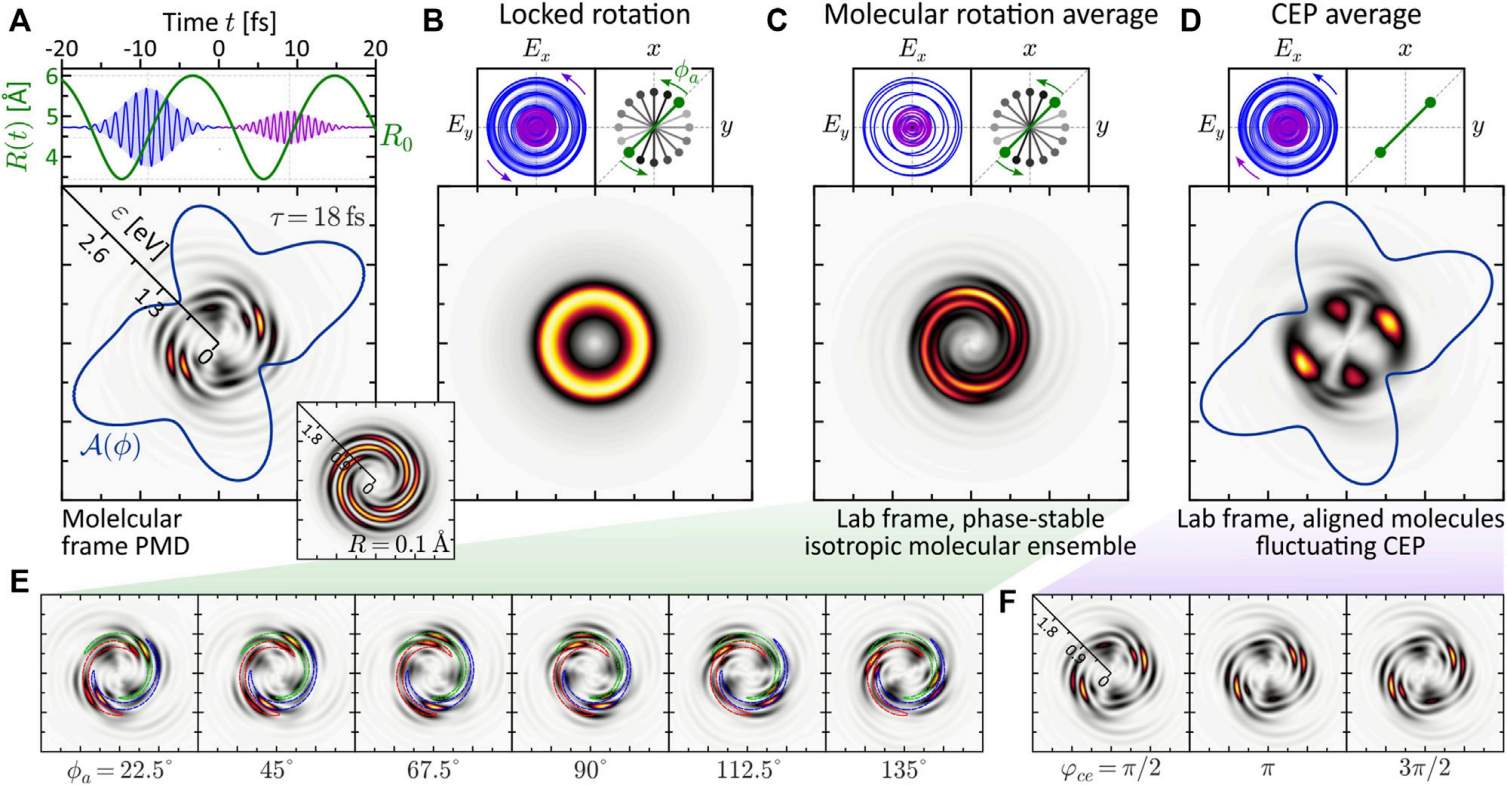
(Color online.) Influence of experimental conditions on a molecular FEV created by dynamic two- vs. three-photon ionization using an LRCP bichromatic (2*ω*: 3*ω*) pulse sequence. All frames show energy-calibrated 2D PMDs. **(A)** The molecular frame PMD created by a phase-stable field exhibits an intricate five-armed spiral pattern. **(B)** Rotating both the molecule and the field about the laser propagation direction washes out the interference pattern completely and yields an isotropic, torus-shaped PMD in the lab frame. **(C)** Lab frame PMD obtained for an isotropic molecular ensemble and a phase-stable pulse sequence. Surprisingly, the highly structured five-armed FEV transforms into a regularly shaped three-armed Archimedean spiral. **(D)** Lab frame PMD obtained for an ensemble of aligned molecules and a fluctuating CEP. The CEP controls the rotation of the bichromatic field. **(E,F)** show selected subsets of molecular frame PMDs underlying the molecular rotation averaging and the CEP-averaging procedure, respectively.

The numerical results are presented in Figure 8. Panel (a) shows the PMD in the molecular frame for a fully stabilized pulse. We observe a structured five-armed FEV consistent with the perturbative ionization model applied to two- vs. three-photon ionization. The FEV is localized in the low-energy region around *ɛ* = 0.75 eV. The reason for the enhancement of the photoelectron signal near the ionization threshold is the resonant excitation of the *ψ*_6_-state by the first subpulse (cf. discussion in Section 3.2.2), which is then mapped into the continuum around *ɛ* = 0.75 eV by absorption of another photon from the second subpulse. The inset shows the PMD obtained in the atomic limit (rigid molecule with *R* = 0.1 Å). Compared to the dynamic case, it displays a more regularly shaped five-armed Archimedean spiral, similar to the result obtained recently for the 2D model of a single potassium atom (Eickhoff et al., 2021c). Figures 8B–D show three types of rotational averaging procedures. In panel (b), both the molecule and the laser field are rotated by 360°, in 16 steps of *δϕ* = 22.5°, keeping their relative orientation locked. In an experiment, this procedure corresponds to a rotation of the detector around an aligned molecule interacting with a phase-stable field. As expected, the average result is an isotropic, torus-shaped PMD which serves as a reference for the other two—experimentally more relevant—averaging studies.

Panel (c) shows the effect of molecular rotation averaging on the PMD. This type of averaging is relevant when measuring on an ensemble of unaligned molecules. In contrast to the real 3D space, where the molecule has two angular degrees of freedom (polar and azimuthal), in 2D we consider the rotation about the azimuthal angle *ϕ*. Due to the reflection symmetry of the homonuclear dimer, only rotations up to an angle of *ϕ*_*a*_ = 180° need to be considered. The alignment angle *ϕ*_*a*_ is taken relative to the positive grid-diagonal in counterclockwise direction. Keeping the laser field fixed, we calculated 16 molecular frame PMDs in which we successively rotated the molecule in steps of *δϕ*_*a*_ = 11.25°. A subset of these calculations is shown in panel (e). Surprisingly, the five-armed spiral pattern is not completely washed out by the molecular rotation, but evolves into a three-armed Archimedean spiral. To rationalize the formation of the three-armed spiral in the lab frame PMD, the series of images in panel (e) illustrates how the maxima of the five-armed spiral shift along a contour of the three-armed spiral of the averaged PMD under the molecular rotation. So far, the exact mechanism behind the counterintuitive transformation of the five-armed spiral into a three-armed spiral upon averaging has not been fully analyzed. Clearly, in order to obtain averaged PMDs that can be compared with experimental results, rotation averaging needs to be performed in 3D. It is quite possible that 3D averaging also blurs other features of the PMD. The results from the 2D model presented here are a natural first step in this direction and serve to compare rotational vs. CEP averaging.

Panel (d) shows the influence of CEP-averaging on the PMD. This type of averaging is especially relevant in bichromatic MPI schemes based on the interference of photoelectron wave packets of different parity (Kerbstadt et al., 2018; Kerbstadt et al., 2019b). In the case of MPI with a bichromatic LRCP sequence, the CEP controls the rotation of the field in the polarization plane and therefore determines the relative orientation between field and molecule. Here, we considered an aligned molecule (*ϕ*_*a*_ = 0) and varied the CEP from *φ*_*ce*_ = 0 to 2*π* in 16 steps of *δφ*_*ce*_ = *π*/8. As a result, the fine five-armed spiral pattern observed in the phase-stable scenario (a) is averaged out. The main lobes of the angular distribution A(ϕ) , however, are maintained because the lobe structure is governed by the molecular structure and alignment rather than the phase of the circularly polarized pulses.

The above results show, that both the molecular alignment and the stability of the optical phase are required for the observation of the fine details characterizing the molecular FEVs. Hence, the retrieval of the rich structural and dynamical information encoded in the PMD from laboratory measurements requires optimal experimental conditions including molecular alignment and phase stability. An alternative to the alignment of molecules is the use of coincidence detection techniques (Ullrich et al., 2003) and post-selection of ionization events corresponding to a specific molecular alignment.

## 4 Summary and Conclusion

Free electron vortices (FEVs) created by photoionization of *atoms* with ultrashort polarization-tailored laser pulse sequences already exhibit a great variety of shapes, as recently reviewed in (Kerbstadt et al., 2019a; Kerbstadt et al., 2020, Eickhoff et al., 2021c). The photoelectron momentum distribution (PMD) of atomic FEVs is characterized by a regularly shaped multi-armed Archimedean spiral (in energy representation) (Pengel et al., 2017b), with controllable symmetry, rotational sense and spiral arm pitch (Kerbstadt et al., 2019b). However, *molecular* FEVs, studied in this contribution, display an even richer structure than their atomic counterparts, resulting from the interplay of three key features. The basic characteristic is the spiral-shaped interference pattern, fully analogous to that of atomic FEVs. This vortex shape is superimposed by a structural feature, due to the broken spherical symmetry of the molecule as opposed to an atom. Finally, a dynamical feature arises due to the nuclear degrees of freedom and the coupling between the electron and nuclear dynamics. The interplay of all these features results in a wide variety of details in the PMD and opens the door to the emerging field of molecular FEVs.

In this numerical study, we have explored the rich structures in the molecular FEVs and studied multiple interference phenomena underlying the ionization dynamics. Our strategy was based on the *ab initio* solution of the 2D time-dependent Schrödinger equation (TDSE) for a diatomic molecular ion with a single active electron, aligned in the polarization plane of a polarization-shaped ultrashort laser pulse. The molecular system was thoroughly characterized by the calculation of the Born-Oppenheimer potentials, eigenfunctions and transition dipole moments. The 2D TDSE model for the light-induced coupled electron-nuclear dynamics was employed to study the PMD created by a number of established laser pulse shapes (Eickhoff et al., 2021a), focusing on the creation of molecular FEVs by counterrotating circularly polarized double pulse sequences. We compared the results from single-photon ionization (SPI) and multiphoton ionization (MPI). The SPI findings were easier to interpret than the MPI results because the latter give rise to multiple ionization pathways (Eickhoff et al., 2021c) and are highly sensitive to intermediate resonances in the bound system. The physical properties of the molecular FEVs were analyzed taking into account various observables, including the fully differential 2D PMD, the (angle-integrated) energy spectrum and the (energy-integrated) angular distribution. In addition, we followed the time evolution of the electron density |*ψ*|^2^ in coordinate space, to reveal the ionization dynamics in the bound molecular system and rationalize the vortex formation dynamics in the continuum. In the investigated MPI schemes, the evaluation of the bound state population dynamics allowed us to unambiguously identify intermediate resonances and unravel their signatures imprinted in the PMD.

Specifically, we presented three interrelated studies. In the first study, we explored the influence of the nuclear configuration on the molecular FEVs. For this purpose, we considered the interaction of a *rigid* molecule with different standard pulse shapes, already used for experiments on MPI of atoms, and analyzed the created PMDs for different internuclear separations *R*. In the limit *R* → 0, the findings reproduced the experimental atomic FEV results and were well-understood in an atomic picture. In the limit *R* → *∞*, complex interference patterns were observed in the PMD, which were created by an interplay of two-center interference and Coulomb diffraction. In the intermediate *R*-regime, around the equilibrium distance *R*_0_, the structure of the PMDs was found to be more intricate and more difficult to explain in a simple picture. This regime was relevant for the second study, in which we considered the interaction of a *vibrating* molecule with specifically designed counterrotating circularly polarized pulse sequences. By synchronization of the two subpulses to the molecular vibration, we investigated the interplay between the nuclear motion and the electronic excitation dynamics and identified signatures of the coupled electron-nuclear dynamics in the PMD of the molecular FEVs. First, we found that the nuclear dynamics result in a bimodal photoelectron kinetic energy spectrum, rationalized by Mullikan’s difference potential analysis. Second, we observed the fingerprint of non-adiabatic phase dynamics of the ground state wave function, which manifested in a subtle but clearly visible radial modulation of the PMD. The latter finding demonstrates the high sensitivity of the PMD to non-adiabatic electron-nuclear dynamics in molecules and underscores the power of highly differential photoelectron detection techniques to reveal even subtle changes in the bound electronic wave function. Further characteristic features of the FEVs created by MPI could be attributed to the influence of two intermediate resonances, which were excited during different stages of the molecular vibration. All these results highlight the great importance of the interplay between the nuclear dynamics and electronic resonances in molecular MPI. The third study was relevant in view of the experimental implementation of molecular FEV scenarios. On the example of bichromatic two- vs. three-photon ionization, we compared the PMD obtained in the molecular frame to the PMD measured in the laboratory which is, in general, subject to different types of averaging. Specifically, we studied the influence of molecular rotation averaging and CEP-averaging. We found that in both cases, some of the fine details of the molecular FEVs were washed out. In order to retrieve the rich structural and dynamical information encoded in the PMD, it is essential to align the molecules prior to photoionization, or detect the momentum of the photoions in coincidence and post-select the events corresponding to a specific molecular alignment, and to stabilize the phase of the laser pulses.

In perspective, our 2D TDSE model will be refined towards the inclusion of quantum mechanical nuclear wave packet dynamics, i.e., a full quantum treatment of the coupled electron-nuclear dynamics. The refined model will allow more accurate simulation of the molecular FEVs generated by MPI in the presence of intermediate resonances. The numerical study presented here provides first insights into the rich variety of relevant physical mechanisms involved in the generation of molecular FEVs with shaped laser pulses and serves as preparatory work for the experimental implementation in the laboratory. The combination of white-light polarization pulse shaping (Pengel et al., 2017a; Kerbstadt et al., 2017b) with photoelectron tomography (Wollenhaupt et al., 2009b; Kerbstadt et al., 2019a) will enable the use of molecular FEVs as powerful spectroscopic tools to probe molecular structure and dynamics.

## Data Availability

The original contributions presented in the study are included in the article/supplementary material, further inquiries can be directed to the corresponding author.
